# Clade density and the evolution of diversity-dependent diversification

**DOI:** 10.1038/s41467-023-39629-5

**Published:** 2023-07-29

**Authors:** Marcio R. Pie, Raquel Divieso, Fernanda S. Caron

**Affiliations:** 1 Biology Department, Edge Hill University, Ormskirk, Lancashire UK; 2 Departamento de Zoologia, Universidade Federal do Paraná, Curitiba, Paraná Brazil

**Keywords:** Macroecology, Evolution, Biodiversity, Speciation

## Abstract

The assumption of an ecological limit to the number of species in a given region is frequently invoked in evolutionary studies, yet its empirical basis is remarkably meager. We explore this assumption by integrating data on geographical distributions and phylogenetic relationships of nearly six thousand terrestrial vertebrate species. In particular, we test whether sympatry with closely-related species leads to decreasing speciation rates. We introduce the concept of clade density, which is the sum of the areas of overlap between a given species and other members of its higher taxon, weighted by their phylogenetic distance. Our results showed that, regardless of the chosen taxon and uncertainty in the phylogenetic relationships between the studied species, there is no significant relationship between clade density and speciation rate. We argue that the mechanistic foundation of diversity-dependent diversification is fragile, and that a better understanding of the mechanisms driving regional species pools is sorely needed.

## Abstract (web summary)

common assumption of evolution is that of an ecological limit to species diversity. This study tests whether sympatry with closely-related species leads to decreasing speciation rates. They find that, for terrestrial vertebrates, the probability of speciation seems to be unaffected by the number of other species of that lineage already present

## Introduction

Is there a limit to the number of species that can coexist in a given region? This question has long intrigued ecologists and evolutionary biologists, dating back at least to Elton^1^, who argued that “…the number of different kinds of animals that can live together in an area of uniform type rapidly reaches a saturation point”. In the following decades, such limits to local diversity became widely accepted, leading to the concept of limiting similarity as a major driver of community structure^2–5^. As a consequence, communities were envisioned as the deterministic outcome of local processes on ecologically homogeneous areas, whereas mechanisms at other spatial and temporal scales were not deemed relevant^3,6^. This framework was well-encapsulated by MacArthur^4^: “… if the areas being compared are not saturated with species, an historical answer involving rates of speciation and length of time available will be appropriate; if the areas are saturated with species, then the answer must be expressed in terms of the size of the niche space… and the limiting similarity of co-existing species.” However, later research would increasingly challenge this view of community structure, especially in three main areas. First, the seemingly widespread occurrence of limiting similarity and constant size ratios appeared to be illusory, as proper statistical analyses commonly failed to provide support for it^7^. In addition, apparently comparable habitats in different regions of the world were shown to harbor dramatically different species richness, such as mangrove^8^ and Mediterranean vegetations^9^. Finally, some studies interpreted asymptotic relationships of local vs. regional diversity as potential evidence for species saturation (e.g., refs. ^10,11^). However, the observation that the properties of local communities could be accurately predicted from regional species pools suggested that the ultimate causes of variation in species richness would actually reflect large-scale evolutionary/historical mechanisms^12^. Moreover, this shift toward larger spatial and temporal scales not only failed to rescue the classical ideas of community saturation, but actually led to two important conundrums. First, if local diversity can be predicted from regional diversity, one would in turn have to explain what mechanisms drive the evolution of regional species pools in the first place. Second, and possibly even more challenging, one would have to reconcile how an inherently local phenomenon (interspecific competition) would translate into regional changes in species pools.

In paleobiology, the issue of diversity limits has been independently explored concurrently with the debates in the ecological literature, and in a similarly contentious manner^13,14^. Some authors have argued that the diversification process is unbounded^15–18^, whereas other authors favored the existence of strong limits to diversity, such that speciation rates would decrease and extinction rates would increase as the number of species in a region approaches its maximum, a phenomenon known as equilibrial dynamics of diversification^19–23^. Three major sources of evidence have been proposed in favor of equilibrial dynamics^24^. First, the concurrent demise of a given clade and proliferation of another ecologically similar taxon has been interpreted as resulting from superior adaptations^25^, such as the substitution of cyclostome bryozoans by cheilostomes^26^, and brachiopods by bivalves (but see refs. ^27–29^). Second, the fossil record of several taxa is characterized by relative stability over long evolutionary timescales, such as North American Cenozoic mammals^30^, Phanerozoic terrestrial vertebrates^23^, and the endemic Cenozoic molluscan fauna of New Zealand^31^. Finally, diversification rates following mass extinctions tend to be considerably higher than normal rates, as one would predict by diversification due to ensuing ecological opportunities (e.g., refs. ^20,32–34^). It is also important to note that, even though these ideas involve negative-diversity dependence in diversification rates, some authors have in fact argued the opposite: as new life forms are continuously being added to a given biota, they would provide new niches, habitats, and potential interactions with other species, such that the overall result could be a positive influence on diversification, i.e., “diversity begets diversity”^17,35,36^. However, one should be cautious when interpreting these results. For instance, palaeobiological analyses traditionally are not based on species, but rather on higher taxonomic levels (but see ref. ^37^), which might not necessarily reflect the same dynamics of the underlying species diversification patterns^38,39^. Apparent patterns of decelerating diversification might result from simple topological constraints in the tree of life, as higher taxonomic levels tend to be described earlier in a given tree^40^, from subjective assessments by taxonomists, and because the origin of higher taxonomic levels conflates phenotypic and lineage diversification, as morphologically distinct clades tend to be recognized more readily as higher taxa than other nodes on a given tree. Indeed, it is important to note that species compete with one another (or, more specifically, individuals in populations of different species), and competition between supraspecific taxa is not a phenomenon that has been properly defined^41^. In addition, most of the data in the fossil record involves invertebrate taxa from the shallow marine shelf, which might not necessarily be representative of terrestrial or other marine environments^38^.

A common element of most efforts to model equilibrial dynamics is the analogy with logistic growth models in population biology. First, the ecological opportunity during the early stages of the diversification of a clade might promote a relatively rapid radiation, both due to lack of competitors and high resource availability^42^. However, as niches become occupied, there would be a corresponding decline in diversification^43^, such that the number of species in a region would be limited by a carrying capacity analogous to the *K* parameter in population biology (e.g., ref. ^43^). With the increasing prevalence of dated phylogenetic hypotheses, such deceleration has been commonly inferred from the temporal branching patterns in phylogenies of extant species (e.g., ref. ^43^., but see refs. ^44,45^), although some studies have emphasized that biases such as misspecified evolutionary models might lead to apparent decelerations (e.g., ref. ^46^). An important limitation of this approach is that, by focusing simultaneously on the total number of species in a region (e.g., refs. ^10,15,19,25^), it implicitly assumes a mean field approximation in which all species are ecologically equivalent, compete identically for resources, and completely overlap their geographical ranges. However, even a cursory view of natural communities immediately makes it obvious that each species has a unique set of range overlaps with other species, of which some might be fierce competitors, whereas sympatry with other species might be completely inconsequential. Therefore, if diversification is indeed limited by competition, and all else being equal, diversification should be inversely proportional to the level of range overlap with other species, weighted by their ecological differences.

**Fig. 1:**
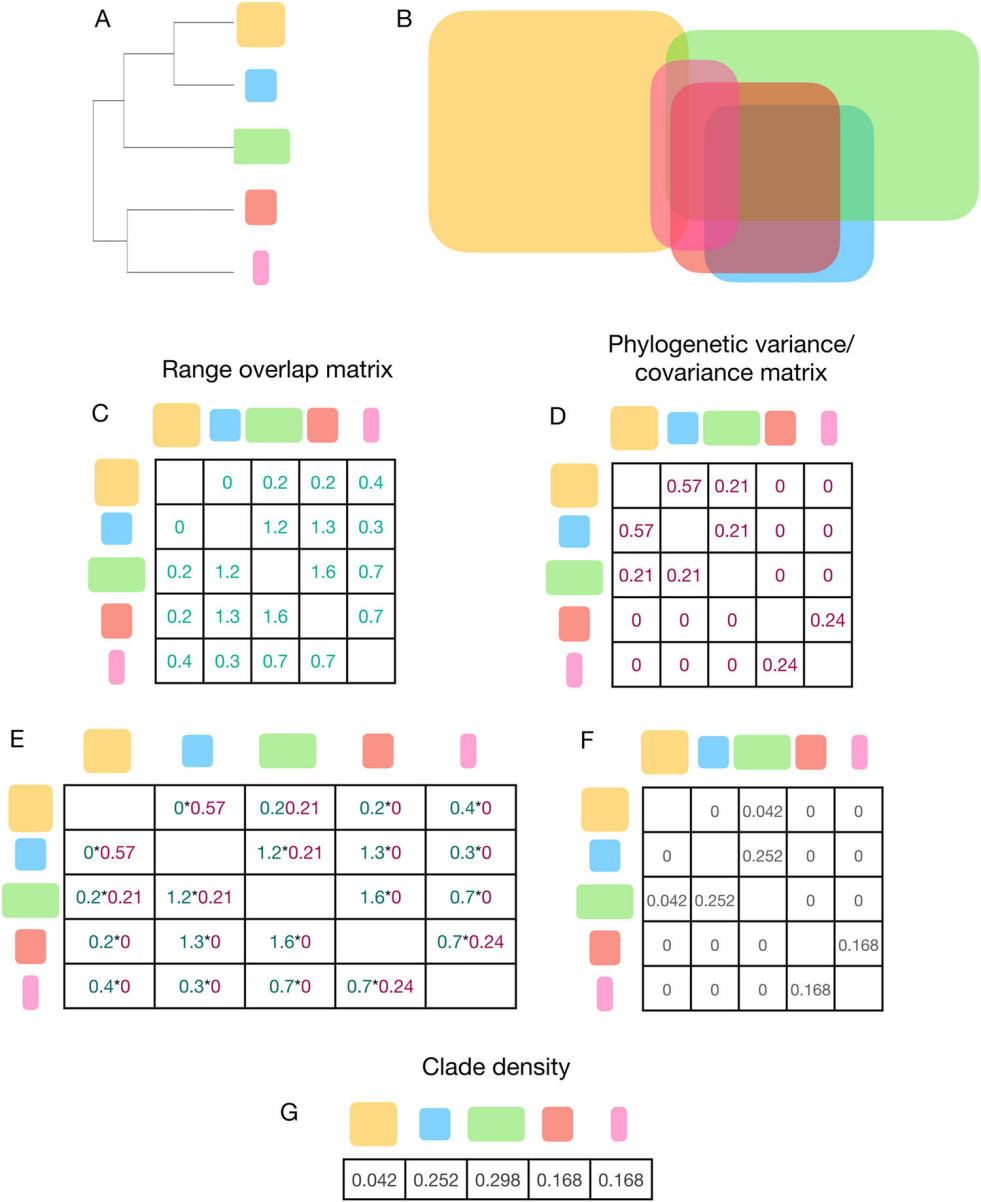
Steps involved in the calculation of clade density. We begin with a set of five species, whose phylogenetic relationships and range sizes are provided in **A** and geographical distributions are shown in **B**. From the geographical distributions, it is possible to obtain a range overlap matrix, which measures the area of overlap between each pair of species (**C**). The phylogeny is then used to calculate the phylogenetic variance-covariance matrix (**D**), which is then multiplied to each element in the range overlap matrix by the phylogenetic variance-covariance (**E**, **F**). All elements in each line are then summed to obtain the estimates of clade density for each species (**G**).

In this study we test a central hypothesis based on expectations from equilibrial dynamics: if diversification is diversity-dependent, the presence of competing species should lower speciation rates. To avoid the mean-field assumption indicated above, we adopted an approach that involves three main steps: (i) we assess the extent to which the geographical distribution of each species in a clade shows overlap with other members of the same higher taxon; (ii) we weigh the level of overlap between each pair of species by its corresponding phylogenetic distance, assuming that their divergence times would provide an indication of the difference in their ecological characteristics. The sum of the weighted range overlaps, hereafter called clade density (Fig. 1), would correspond to the level of potential ecological influence on a given species by other members of its higher taxon; and (iii) we test the extent to which clade density can predict variation in speciation rates of four large clades of terrestrial vertebrates.

## Results

**Fig. 2:**
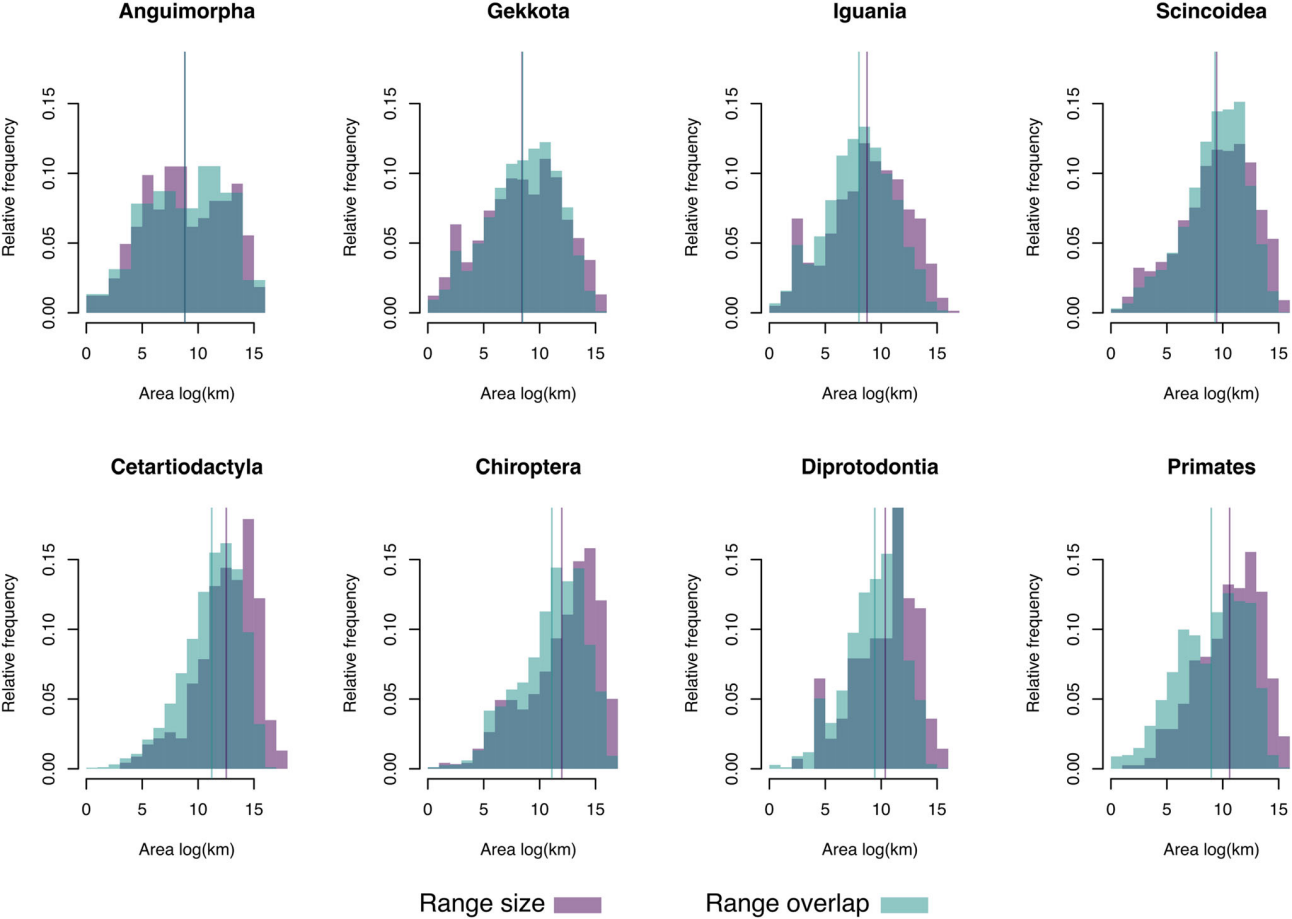
Frequency distributions of range sizes and range overlap sizes for different terrestrial vertebrate groups. Range overlap sizes were calculated for all pairs of species in each taxon. Vertical lines indicate the means of the corresponding log-transformed data. Source data are provided as a Source Data file.

**Fig. 3:**
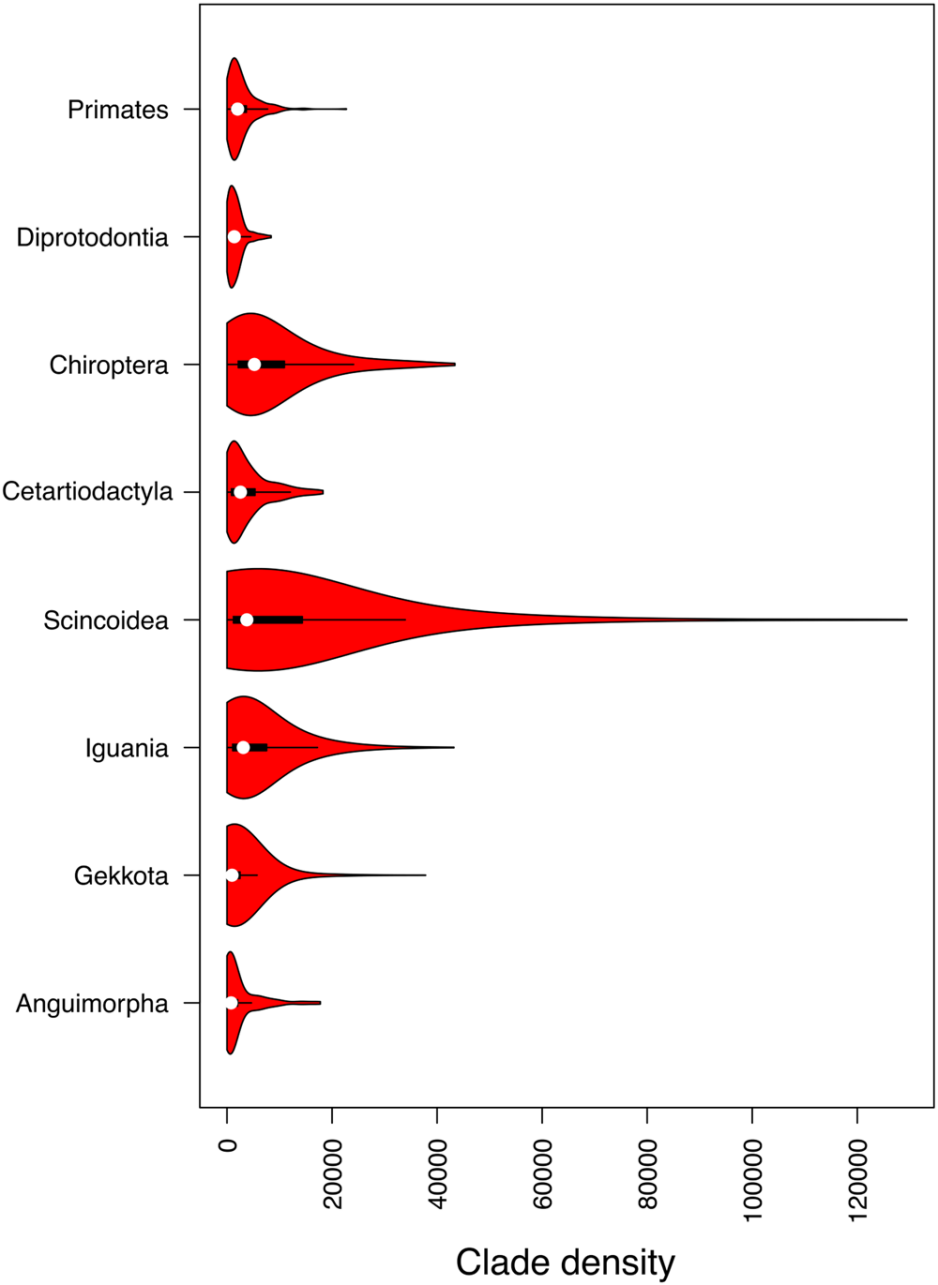
Violin plots of clade densities across species in different terrestrial vertebrate groups. Distributions are invariably asymmetric, with most species showing relatively low values of clade density. The number of species in each clade is as follows: Anguimorpha [*N* = 162], Gekkota [*N* = 1225], Iguania [*N* = 1395], Scincoidea [*N* = 1216], Cetartiodactyla [*N* = 230], Chiroptera [*N* = 1182], Diprotodontia [*N* = 139], and Primates [*N* = 387]. Data are presented as: Anguimorpha [min = 0; lower whisker=0; 25th percentile = 261.134; median = 778.52; 75th percentile = 2013.554; upper whisker = 4642.184; max = 17754.086], Gekkota [min = 0; lower whisker = 0; 25th percentile = 297.563; median = 954.457; 75th percentile = 2469.793; upper whisker = 5728.137; max = 37806.053], Iguania [min = 0; lower whisker = 0; 25th percentile = 1089.367; median = 3097.616; 75th percentile = 7538.88; upper whisker = 17213.15; max = 43206.41], Scincoidea [min = 0; lower whisker = 0; 25th percentile = 1239.11; median = 3777.291; 75th percentile = 14321.733; upper whisker = 33945.67; max = 129425.128], Cetartiodactyla [min = 0; lower whisker = 0; 25th percentile = 859.038; median = 2556.897; 75th percentile = 5330.742; upper whisker = 12038.3; max = 18265.094], Chiroptera [min = 0; lower whisker = 0; 25th percentile = 2148.482; median = 5211.879; 75th percentile = 10922.92; upper whisker = 24084.58; max = 43382.571], Diprotodontia [min = 0; lower whisker = 0; 25th percentile = 653.209; median = 1377.411; 75th percentile = 2197.731; upper whisker = 4514.512; max = 8386.167], and Primates [min = 0; lower whisker = 0; 25th percentile = 973.24; median = 2016.426; 75th percentile = 3676.591; upper whisker = 7731.617; max = 22711.306]. Source data are provided as a Source Data file.

**Fig. 4:**
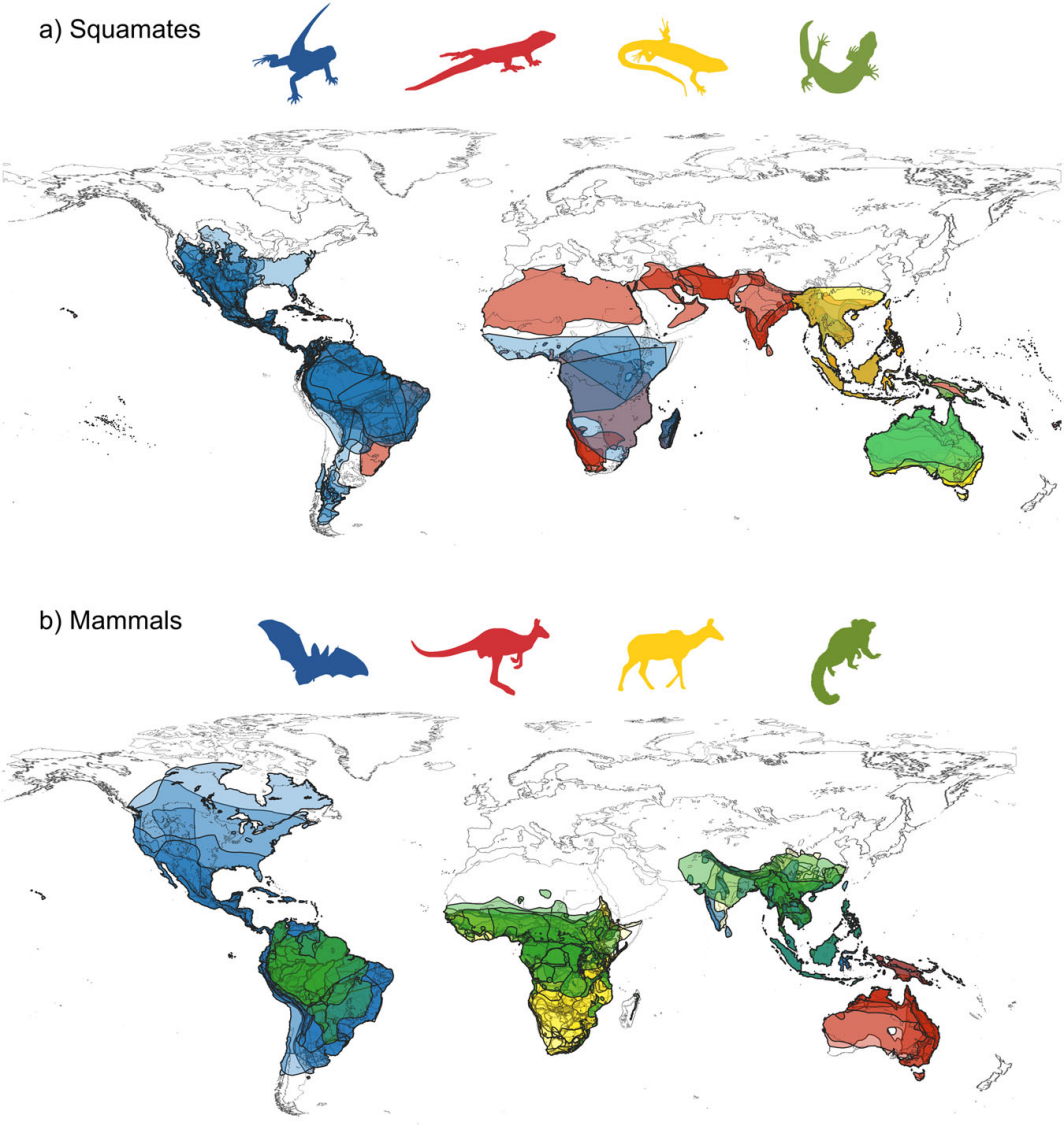
Global distribution of 10% of the species with the highest clade density value in each analyzed clade. More intense colors indicate a concentration of overlapping ranges. In the case of squamates (**a**), the analyzed clades include: Iguania (blue), Gekkota (red), Scincoidea (yellow), and Anguimorpha (green). For mammals (**b**), the analyzed clades consist of: Chiroptera (blue), Diprotodondia (red), Cetartiodactyla (yellow), and Primates (green). The silhouette images were available under Public Domain license at PhyloPic^81^. Maps were generated in QGIS^82^ (version 3.22.3) using range maps obtained from IUCN^71^.

The frequency distributions of range sizes and range overlap sizes for each taxon are provided in Fig. 2. In general, there was broad correspondence between the shape of these distributions, with the frequency distribution of range overlap sizes being slightly shifted towards the left. Therefore, based on these results, one could naively expect that clade densities would be high across species in all groups. However, the distribution of clade density estimates was invariably skewed, with the vast majority of species showing low values and only a relatively small number of species living in conditions of high clade density (Fig. 3). In other words, although range overlap is common, conditions in which a species overlaps with many closely-related lineages is rare. The geographical distribution of species with unusually high clade densities are shown in Fig. 4. Regions with squamate species with high clade density were largely incongruent between Anguimorpha, Gekkota, Iguania, and Scincoidea, reflecting distinct preferences of each taxon for different types of biomes (Fig. 4a). On the other hand, mammal species with high clade density tended to be most common in the humid tropics worldwide – except for Diprotodontia, which was concentrated in Oceania for biogeographical reasons (Fig. 4b). Interestingly, all chiropterans with high clade density were found in the New World, spanning most of the distribution of bats in the continent, whereas no Old World bat was found within the top 10% of the species with highest clade densities.

**Fig. 5:**
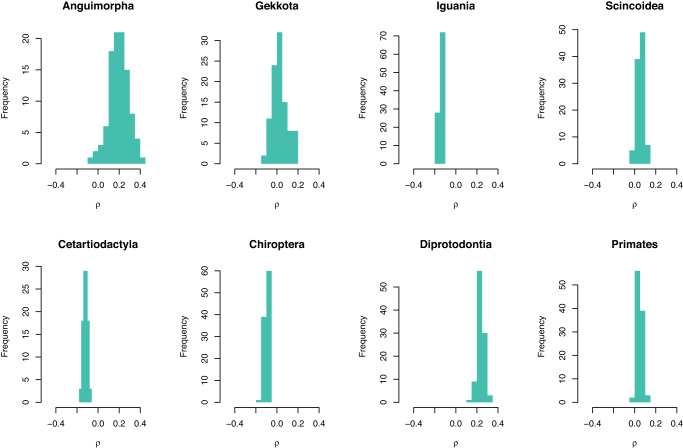
Frequency distribution of the slopes of relationships between variation in clade density and speciation rate (λDR). Distribution is based on different tested topologies in each taxon. Source data are provided as a Source Data file.

Given the considerable variation in speciation rates among species of the studied taxa, as measured by their corresponding λDR statistic (Fig. S1), there should be sufficient statistical power to detect the influence of clade density on speciation rates. However, after taking into account phylogenetic uncertainty, the association between speciation rates and clade densities varied between slightly positive to slightly negative in different groups depending on the particularly chosen topology (Fig. 5). In particular, regardless of the taxon, and despite the inherent phylogenetic uncertainty, none of these associations were statistically significant for any of the tested topologies, supporting the conclusion that speciation rates are unaffected by the observed levels of clade density.

## Discussion

In this study we introduce the concept of clade density and use it to test predictions of equilibrial models of diversification. Contrary to expectations, we showed that (i) clade density varies considerably between species, and it is only high in very few of them; and (ii) there is no relationship between clade density and speciation rates. These results bring into question the generality of models of diversity-dependent diversification, at least for terrestrial vertebrates. Our conclusions disagree with some recent studies on equilibrial dynamics. For instance, Weir^47^ found a significant relationship between decelerating lineage accumulation and the maximum number of regionally sympatric species of Neotropical birds, suggesting that speciation rates declined as the number of species increased. However, this effect was only detected for lowland, but not for highland clades. Machac et al. ^48^ have shown that the strength of diversification slowdown in mammalian clades is related to the overlap of species ranges within clades, so that clades with many overlapping ranges reveal more pronounced slowdowns (but see ref. ^49^). Likewise, Kennedy et al. ^50^ have shown that diversification slowdown in bird clades is related to species range overlap, and the clades not revealing slowdowns are those that expand geographically or in functional space.

One important limitation of these studies is that they rely on the γ-statistic^51^, which estimates whether the nodes within a phylogeny are disproportionately distributed toward the root or tips of the tree. Therefore, due to the nature of the method, the γ-statistic tends to give more emphasis to the deep nodes of the tree, whereas changes in terminal nodes (for which one would have more precise information on species distributions) are less consequential. In addition, the γ-statistic is strongly affected by the arbitrary decision of which node and descending branches represent the clade to be studied, given that adding even one more inclusive node could strongly affect the γ-statistic. Even more worryingly, it assumes that all species equally affect one another—even those that are completely allopatric. On the other hand, the clade density approach proposed here has two important and desirable properties: it disproportionately weighs closely related lineages (which are thought to show the highest potential to exert interspecific competition), and it only allows for species to be subject to interspecific competition if they show some level of sympatry. As a consequence, clade density is a particularly suitable statistic to detect diversity-dependent diversification, if these were indeed present and driven by competition between sympatric relatives. In particular, if environmental variables, such as temperature and productivity, were important drivers of an interspecific regional carrying capacity, one would find concordant patterns in the geographical distribution of lineages with high clade density. Although mammal species under high clade density were indeed particularly concentrated in the humid tropics (Fig. 4C), the highly disjunct distribution of high clade density lineages of squamates suggests that these patterns might simply reflect historical factors such as niche conservatism, as opposed to areas of consistently high interspecific regional carrying capacity. One interesting observation revealed by using this metric is the detection of a relatively small subset of lineages living under substantially higher clade density. These lineages could potentially represent hotspots of (co)evolutionary dynamics (e.g., as particular hubs for host-parasite host shifts), and seem like ideal model system to explore the evolutionary consequences of variation in clade density (see also ref. ^52^).

There are six important caveats that should be kept in mind when interpreting our results. First, geographical range data are notoriously imprecise, particularly regarding errors in determining range boundaries. Although this is a necessary limitation of studies at this scale, we believe that this is unlikely to lead to systematic bias that would change our conclusions, given that the vertebrate taxa included in our analyses are among the animal lineages with the best known geographical distributions. Second, geographical ranges are dynamic both in space (a species might not be equally distributed throughout the available distribution polygons) and time (ranges might expand and contract following regional climatic fluctuations). Fully accounting for this variation is challenging, except for a small minority of species with good geographical and fossil data. However, inhomogeneous distributions across space and time seem more likely to lead to lower opportunities for sympatry, such that our results could be seen as conservative. Third, our use of the λDR statistic means that our results reflect differences in speciation rates, whereas equilibrial dynamics could presumably be related to extinction rates instead. Estimating extinction rates with extant lineages alone is a notoriously difficult task, but Pires et al. ^53^ provided evidence to indicate that equilibrial dynamics within a clade should primarily affect speciation rates (see also refs. ^54–56^). Moreover, counter-acting positive diversity-dependence could potentially obscure the signal of diversity dependence in the analyses presented here. Fourth, species concepts and taxonomic traditions might change across different studied taxonomic groups. For instance, if a given taxon tends to be classified under “splitter” or “lumper” traditions, that will affect how their geographical distributions and divergence times are computed. Although these effects should be explored in future studies, the fact that we see consistently negative results across all taxa suggests that this factor might not be strong enough to bias our conclusions. Fifth, we assume that species with non-overlapping ranges do not influence their per-lineage diversification rates. For instance, one could envision a scenario in which the interaction between two competing species leads to complete competitive exclusion, so that their distributions are completely parapatric. Although possible, we believe that this mechanism would be unlikely to affect our conclusions for three main reasons: (1) well-documented cases of parapatry due to competitive interactions are exceedingly rare and often involve alternative hypotheses (e.g., sharp ecotones); (2) the effect on diversification would involve the resulting decrease in geographical range, yet the relationship between species richness and range size is far from well-established; and (3) whatever the potential impact of allopatric species, it seems that it would be considerably weaker than that of co-existing species. Finally, our measure of clade density assumes that there is a direct relationship between ecological and phylogenetic distances between species. The idea that closely-related species tend to compete more strongly dates back at least to Darwin, but its generality is often questioned (e.g., ref. ^57^.). Future studies that explicitly incorporate functional differences between lineages could provide valuable insight into the potential levels of interspecific competition in a framework similar to that developed here to measure clade density, particularly in cases where diversity-dependence might not scale proportionally with phylogenetic distance.

The dichotomy between diversity-independent and diversity-dependent drivers of species diversification has direct parallels with a similar controversy in population biology^25^. Following the pioneering ideas by Elton^58^, Lotka^59^, and Volterra^60^, explanations for population fluctuations were framed as being due to exogenous (climatic) variables, as opposed to endogenous (density-dependent) factors^61,62^. As in the study of population biology, this debate has dragged on for decades^63,64^ due to the scarcity of methodological and conceptual tools that could clearly distinguish predictions of either model. In particular, a geographical component of lineage diversification is often missing^65^, even though the level of sympatry has been argued as reflecting the extent to which a region is saturated^66^. In this study we sought to bridge this gap by introducing the concept of clade density and found no evidence for equilibrial dynamics in two species-rich and ecologically diverse groups of terrestrial vertebrates. The consistent lack of association between clade density and diversification behooves us to re-think the conceptual basis of equilibrial dynamics to begin with. It is predicated on the phenomenon of interspecific competition, which is indeed prevalent, but then tacitly makes a number of leaps in argument, positing that interspecific competition between sets of species at local scales translates into a consistent effect at regional scales, which simultaneously and equally impacts all species in a given taxon (even if they are not sympatric), to such an extent that it leads to a diversity-dependent depression in diversification rates. Given that there does not seem to be a direct relationship between standing diversity and diversification rates (e.g., refs. ^49,67^), the empirical support for most of those links is tenuous at best. The increased availability of large-scale phylogenetic and biogeographical datasets should allow for unprecedented opportunities to assess mechanisms driving regional diversities, but such assessments should also be accompanied by more rigorous framing of the underlying assumptions of the models being tested.

## Methods

### Data collection

Phylogenetic relationships in this study were obtained from the PHYLACINE 1.2.1 database for mammals^68^ and from Tonini et al.^69^ for squamates. These groups were chosen both because they are among the terrestrial organisms with the best phylogenetic and geographical information available, but also because ecto- and endotherms seem to represent qualitatively distinct modes of geographical range evolution^70^. Instead of including all of the species in those taxa, we studied subclades that were more ecologically homogeneous to facilitate the interpretation of the obtained results, focusing on the particular species for which both phylogenetic and distribution data were available. The combined dataset included 5936 species distributed across mammals and squamates. The number of species included in this study and the corresponding value of the total estimated percentage of species in each clade for mammals are: Cetartiodactyla [*N* = 230, 69%], Chiroptera [*N* = 1,182, 88%], Diprotodontia [*N* = 139, 94%], Primates [*N* = 387, 76%], and for squamates are: Anguimorpha [*N* = 162, 65%], Gekkota [*N* = 1,225, 54%], Iguania [*N* = 1,395, 67%], and Scincoidea [*N* = 1,216, 64%]. Geographical distributions were obtained from shapefiles available on The IUCN Red List of Threatened Species database Version 2018-2^71^. We used the cylindrical equal area projection to minimize systematically overestimating areas near the poles. All uncertain and introduced ranges classified by IUCN criteria were not included in our analysis. Using the IUCN polygons of all available species in each group of vertebrates, we calculated the area of range overlap between all pairs of species and the range size of each species using the ‘gArea’ and ‘gIntersection’ functions from ‘rgeos’ 0.5–9^72^. All analyses in this paper were carried out in R 4.2.1^73^.

### Measuring clade density

Several approaches have been used in previous work to measure the extent of geographical overlap between a set of species. For instance, Weir^47^ used the maximum number of regionally sympatric species as a measure of the extent of co-occurrence, which provides a useful upper limit to the level of range overlap, but that would require cautious interpretation, given that such level of range overlap might not represent the typical conditions found across the range of most species, as some species might only overlap in the periphery of their range. Alternatively, Kennedy et al. ^50^ used the mean number of co-occurring species across all distributions. In this study we introduce a metric called clade density, which measures the extent to which a given species is sympatric with other lineages, weighted by their corresponding phylogenetic similarity. An illustration of the steps involved in the calculation of clade density is provided in Fig. 1 and explained below. We begin with a set of five species, whose phylogenetic relationships and range sizes are indicated in Fig. 1A and geographical distributions are shown in Fig. 1B. We then calculate a range overlap matrix, which measures the area of overlap between each pair of species (Fig. 1C). Using the phylogeny, we calculate the phylogenetic variance-covariance matrix (Fig. 1D) and then multiply each element in the range overlap matrix by the phylogenetic variance-covariance (Fig. 1E, F). Finally, we sum all of the elements in each line to obtain the estimates of clade density for each species (Fig. 1G). It is important to note that we did not account for species for which there was no geographical information in our estimates of clade density, as there was no obvious means to do it objectively and systematically. However, given that those species tend to be poorly known taxa with small ranges, they are unlikely to greatly affect estimates of clade density.

### Estimating speciation rates

We explored variation in speciation rates using the DR statistic^74^ (hereafter, λDR). λDR is a non-model-based estimator of speciation rate that is calculated as a weighted average of the inverse branch lengths between a given species to the root of the phylogeny (i.e., the root-to-tip set of branches). It is therefore similar to the node-density estimator^75^, except that it places more emphasis on recent branch lengths^74^. As a consequence, λDR tends to reflect speciation rates rather than net diversification rates^74,76^. It is important to note that the relationship between clade density and λDR is not obvious, given that one can envision a scenario with high speciation rates and zero clade densities if all species are allopatric, whereas one could find relatively low speciation rates and high clade density if all species are sympatric. In our analyses, speciation rates were calculated based on all species in the original phylogenies, as opposed to only the species with information on geographical ranges, so as not to bias them downward.

We tested for an association between speciation rates and clade density using ‘ES-sim’ (available at https://github.com/mgharvey/ES-sim), which is a semi-parametric test for trait-dependent diversification analyses^77^. In this approach, instead of explicitly modeling the relationship between candidate traits and diversification, the analyses test for correlations between summary statistics of phylogenetic branching patterns and trait variation at the tips of a given phylogenetic tree. The use of tip-specific metrics of speciation rate has been recently suggested as an alternative to parametric state-dependent diversification due to the elevated rates of false-positive results, given that heterogeneity in diversification rates of the underlying phylogeny could bias inferences of associations between traits and diversification regardless of their underlying relationship^78^. Simulations have shown that the use of ES-sim for continuous traits provides equal or superior power than QuaSSE^77^. In addition, given that they are computationally fast, the use of tip-specific metrics makes it feasible to explore the impact of phylogenetic uncertainty in the analyses. ES-sim was implemented using the code provided by Harvey & Rabosky^77^ (available at https://github.com/mgharvey/ES-sim), with 100 simulations used to build the null distribution of trait-speciation associations for significance testing. As the choice of phylogenetic data plays a major role in large-scale inferences of diversification patterns^79^, we accounted for phylogenetic uncertainty by repeating each analysis for 100 alternative topologies.

### Geographical variation in clade density

Given that there was considerable variation in clade density across species (see Results), we also explored spatial variation in clade density. We selected all species within the top 10% clade densities within its taxon and plotted their geographical distributions in a global map. We then qualitatively looked for geographical congruence across taxa, which would be indicative of common underlying mechanisms.

### Reporting summary

Further information on research design is available in the Nature Portfolio Reporting Summary linked to this article.

## Supplementary information

Supplementary Information

Reporting Summary

## Data Availability

All data used in our analyses is available for public access, and the specific sources are provided in the corresponding sections of our methods. In particular, phylogenies were obtained from the PHYLACINE 1.2.1 database for mammals^68^ and from Tonini et al.^69^ for squamates and geographical distribution data were obtained from the IUCN Red List of Threatened Species database Version 2018-2^71^. Source data are provided as a Source Data file. Source data are provided with this paper.

## Source data

Source data

## Code availability

All codes developed for our analyses are available in a public repository^80^ (https://github.com/raqueldivieso/clade_density).
