# Supplementary material for: Clade density and the evolution of diversity-dependent diversification
Source: Nat Commun. 2023 Jul 29;14:4576. doi: 10.1038/s41467-023-39629-5 (PMC10387094; doi:10.1038/s41467-023-39629-5)
Supplement: Supplementary file 1 — Supplementary Information [file 41467_2023_39629_MOESM1_ESM.pdf]

Supplementary Information

Clade density and the evolution of diversity-dependent diversification

Marcio R. Pie[1*], Raquel Divieso[2] and Fernanda S. Caron[2]

[1] Biology Department, Edge Hill University, Ormskirk, Lancashire, United Kingdom.

[2] Departamento de Zoologia, Universidade Federal do Paraná, Curitiba, Paraná, Brazil.

[*]Author for correspondence.

Address for correspondence: Biology Department, Edge Hill University, Ormskirk, Lancashire, L39 4QP, United Kingdom. Email: piem@edgehill.ac.uk

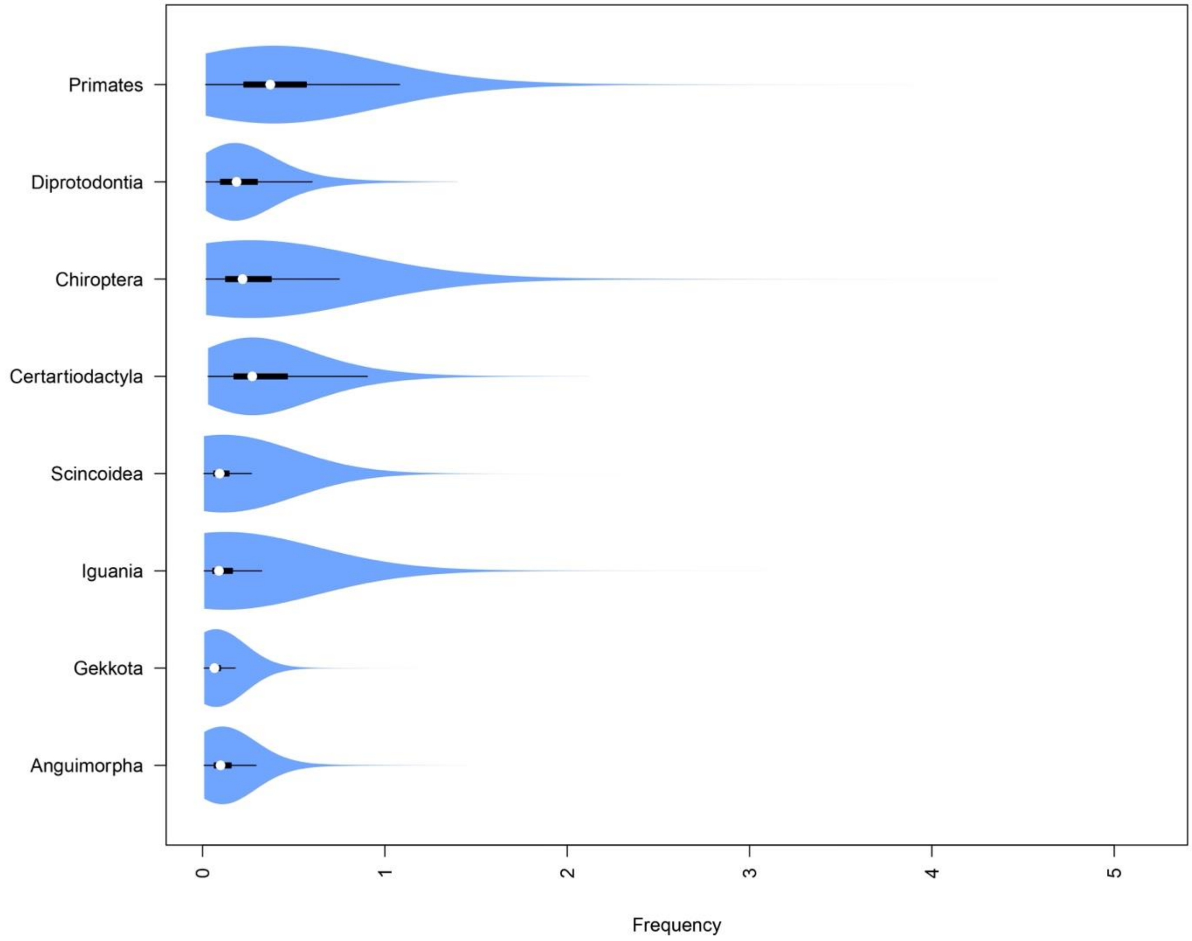

Figure S1. Variation in speciation rates, as measured by the λDR statistic, in the studied taxa. See text for details. The number of species in each clade for are as follows: Anguimorpha [N=162], Gekkota [N=1,225], Iguania [N=1,395], Scincoidea [N=1,216], Cetartiodactyla [N=230], Chiroptera [N=1,182], Diprotodontia [N=139], and Primates [N=387]. Data are presented as: Anguimorpha [min=0.009; lower whisker=0.009; 25th percentile=0.065; median=0.099; 75th percentile=0.156; upper whisker=0.293; max=1.469], Gekkota [min=0.009; lower whisker=0.009; 25th percentile=0.044; median=0.065; 75th percentile=0.097; upper whisker=0.178; max=1.382], Iguania [min=0.009; lower whisker=0.009; 25th percentile=0.056; median= 0.09; 75th percentile=0.163; upper whisker=0.3235; max=5.156], Scincoidea [min=0.007; lower whisker=0.007; 25th percentile=0.062; median=0.093; 75th percentile=0.144; upper whisker=0.268; max=3.985], Cetartiodactyla [min=0.03; lower whisker=0.03; 25th percentile=0.173; median=0.272; 75th percentile=0.465; upper whisker=0.903; max=2.116], Chiroptera [min=0.019; lower

whisker=0.019; 25th percentile=0.127; median=0.219; 75th percentile=0.376; upper whisker=0.75; max=5.195], Diprotodontia [min=0.018; lower whisker=0.018; 25th percentile=0.099; median=0.186; 75th percentile=0.3; upper whisker=0.6; max=1.399], and Primates [min=0.017; lower whisker=0.017; 25th percentile=0.227; median=0.371; 75th percentile=0.569; upper whisker=1.081; max=4.85].
