# Supplementary material for: Clade density and the evolution of diversity-dependent diversification
Source: Nat Commun. 2023 Jul 29;14:4576. doi: 10.1038/s41467-023-39629-5 (PMC10387094; doi:10.1038/s41467-023-39629-5)
Supplement: Supplementary file 2 — Reporting Summary [file 41467_2023_39629_MOESM2_ESM.pdf]

nature portfolio

Corresponding author(s): Marcio R. Pie

Last updated by author(s): Mar 14, 2023

Reporting Summary

Nature Portfolio wishes to improve the reproducibility of the work that we publish. This form provides structure for consistency and transparency in reporting. For further information on Nature Portfolio policies, see our Editorial Policies and the Editorial Policy Checklist.

Statistics

For all statistical analyses, confirm that the following items are present in the figure legend, table legend, main text, or Methods section.

n/a	Confirmed	
☐	☒	The exact sample size (n) for each experimental group/condition, given as a discrete number and unit of measurement
☐	☒	A statement on whether measurements were taken from distinct samples or whether the same sample was measured repeatedly
☒	☐	The statistical test(s) used AND whether they are one- or two-sided<br>*Only common tests should be described solely by name; describe more complex techniques in the Methods section.*
☐	☒	A description of all covariates tested
☐	☒	A description of any assumptions or corrections, such as tests of normality and adjustment for multiple comparisons
☐	☒	A full description of the statistical parameters including central tendency (e.g. means) or other basic estimates (e.g. regression coefficient) AND variation (e.g. standard deviation) or associated estimates of uncertainty (e.g. confidence intervals)
☒	☐	For null hypothesis testing, the test statistic (e.g. F, t, r) with confidence intervals, effect sizes, degrees of freedom and P value noted<br>*Give P values as exact values whenever suitable.*
☒	☐	For Bayesian analysis, information on the choice of priors and Markov chain Monte Carlo settings
☒	☐	For hierarchical and complex designs, identification of the appropriate level for tests and full reporting of outcomes
☒	☐	Estimates of effect sizes (e.g. Cohen's d, Pearson's r), indicating how they were calculated

Our web collection on statistics for biologists contains articles on many of the points above.

Software and code

Policy information about availability of computer code

Data collection	All analyses in this paper were carried out in R 4.2.1, with the packages 'ape' 5.7.1, 'bigmemory' 4.6.1, 'dplyr' 1.1.1, 'geiger' 2.6.11, 'phytools' 1.5.1, 'rgdal' 1.6.5, 'rgeos' 0.6.2, 'stringr' 1.5.0, 'tidyr' 1.3.0, and extra codes from https://raw.githubusercontent.com/mgharvey/ES-sim/master/R/essim.R.
Data analysis	All codes developed for our analyses are available in a public repository (https://github.com/raqueldivieso/clade_density).

For manuscripts utilizing custom algorithms or software that are central to the research but not yet described in published literature, software must be made available to editors and reviewers. We strongly encourage code deposition in a community repository (e.g. GitHub). See the Nature Portfolio guidelines for submitting code & software for further information.

Data

Policy information about availability of data

All manuscripts must include a data availability statement. This statement should provide the following information, where applicable:

- Accession codes, unique identifiers, or web links for publicly available datasets
- A description of any restrictions on data availability
- For clinical datasets or third party data, please ensure that the statement adheres to our policy

Two types of (open access) data were used in this study: geographical distribution data and phylogenetic data. The geographical polygons were obteined from IUCN

database (https://www.iucnredlist.org/.) The phylogenies of: Faurby, S. et al. PHYLACINE 1.2: The Phylogenetic Atlas of Mammal Macroecology. Ecology 99, 2626–2626 (2018) and Tonini, J. F. R., Beard, K. H., Ferreira, R. B., Jetz, W. & Pyron, R. A. Fully-sampled phylogenies of squamates reveal evolutionary patterns in threat status. Biol. Conserv. 204, 23–31 (2016).

Human research participants

Policy information about studies involving human research participants and Sex and Gender in Research.

Reporting on sex and gender	N/A
Population characteristics	N/A
Recruitment	N/A
Ethics oversight	N/A

Note that full information on the approval of the study protocol must also be provided in the manuscript.

Field-specific reporting

Please select the one below that is the best fit for your research. If you are not sure, read the appropriate sections before making your selection.

☐ Life sciences ☐ Behavioural & social sciences ☒ Ecological, evolutionary & environmental sciences

For a reference copy of the document with all sections, see nature.com/documents/nr-reporting-summary-flat.pdf

Ecological, evolutionary & environmental sciences study design

All studies must disclose on these points even when the disclosure is negative.

Study description	In this study we use available data for 19256 vertebrate species distribution and phylogenetic relationships to produce pair species matrix of geographical overlap ranges and phylogenetic distances. We test whether sympatry with closely-related species might decrease speciation rates, and/or slow the attainment of secondary sympatry in sister species.
Research sample	Two types of (open access) data were used in this study: geographical distribution data and phylogenetic data. The geographical polygons were obteined from IUCN database (https://www.iucnredlist.org/.) The phylogenies of: Faurby, S. et al. PHYLACINE 1.2: The Phylogenetic Atlas of Mammal Macroecology. Ecology 99, 2626–2626 (2018) and Tonini, J. F. R., Beard, K. H., Ferreira, R. B., Jetz, W. & Pyron, R. A. Fully-sampled phylogenies of squamates reveal evolutionary patterns in threat status. Biol. Conserv. 204, 23–31 (2016). Squamates and mammals were chosen both because they are among the terrestrial organisms with the best phylogenetic and geographical information available, but also because ecto- and endotherms seem to represent qualitatively distinct modes of geographical range evolution. Instead of including all of the species in those taxa, we studied subclades that were more ecologically homogeneous to facilitate the interpretation of the obtained results, focusing on the particular species for which both phylogenetic and distribution data were available. The combined dataset included 19,256 species, more specifically: the number of species in each clade for mammals are: Cetartiodactyla [N=230], Chiroptera [N=1,182], Diprotodontia [N=139], and Primates [N=387], and for squamates are: Anguimorpha [N=162], Gekkota [N=1,225], Iguania [N=1,395], and Scincoidea [N=1,216].
Sampling strategy	For analysis we use all species for both clades with geographical ranges available in IUCN dataset and that were present in the cited phylogenies. We use only IUCN data to maintain standardization of ranges sizes and locations estimation across all species.
Data collection	RD and FSC were responsible for the data collection. All analyses in this paper were carried out in R 4.2.1, with the packages 'ape' 5.7.1, 'bigmemory' 4.6.1, 'dplyr' 1.1.1, 'geiger' 2.6.11, 'phytools' 1.5.1, 'rgdal' 1.6.5, 'rgeos' 0.6.2, 'stringr' 1.5.0, 'tidyr' 1.3.0, and extra codes from https://raw.githubusercontent.com/mgharvey/ES-sim/master/R/essim.R.
Timing and spatial scale	The geographical range were obteined from IUCN version 2018-2 in february of 2019.
Data exclusions	There was no exclusion of data from the analyses.
Reproducibility	The entire analysis process can be reproduced with the codes provided.
Randomization	Randomization was not a part of our study, considering we did not performed analyses with experimental groups.
Blinding	Blinding was not relevant for this study because information about taxonomic groups didn't have the potential to influence study results.

Did the study involve field work? ☐ Yes ☒ No

Reporting for specific materials, systems and methods

We require information from authors about some types of materials, experimental systems and methods used in many studies. Here, indicate whether each material, system or method listed is relevant to your study. If you are not sure if a list item applies to your research, read the appropriate section before selecting a response.

Materials & experimental systems

n/a	Involved in the study
☒	☐ Antibodies
☒	☐ Eukaryotic cell lines
☒	☐ Palaeontology and archaeology
☒	☐ Animals and other organisms
☒	☐ Clinical data
☒	☐ Dual use research of concern

Methods

n/a	Involved in the study
☒	☐ ChIP-seq
☒	☐ Flow cytometry
☒	☐ MRI-based neuroimaging
